# Toxicity Evaluation following Intratracheal Instillation of Iron Oxide in a Silica Matrix in Rats

**DOI:** 10.1155/2014/134260

**Published:** 2014-05-14

**Authors:** Alina Mihaela Prodan, Carmen Steluta Ciobanu, Cristina Liana Popa, Simona Liliana Iconaru, Daniela Predoi

**Affiliations:** ^1^Emergency Hospital Floreasca, Bucharest 5, 8 Calea Floreasca, Sector 1, 014461 Bucharest, Romania; ^2^National Institute of Materials Physics, 105 Bis Atomistilor, 077125 Magurele, Romania; ^3^Faculty of Physics, University of Bucharest, 405 Atomistilor, 077125 Magurele, Romania; ^4^ISTO, UMR 7327 CNRS, Université d'Orléans, 1A rue de la Férollerie, 45071 Orléans Cedex 2, France

## Abstract

Iron oxide-silica nanoparticles (IOSi-NPs) were prepared from a mixture of ferrous chloride tetrahydrate and ferric chloride hexahydrate dropped into a silica xerogel composite. The structure and morphology of the synthesized maghemite nanoparticles into the silica xerogel were analysed by X-ray diffraction measurements, scanning electron microscopy equipped with an energy dispersive X-ray spectrometer, and transmission electron microscopy. The results of the EDAX analysis indicated that the embedded particles were iron oxide nanoparticles. The particle size of IOSi-NPs calculated from the XRD analysis was estimated at around 12.5 nm. The average size deduced from the particle size distribution is 13.7 ± 0.6 nm, which is in good agreement with XRD analysis. The biocompatibility of IOSi-NPs was assessed by cell viability and cytoskeleton analysis. Histopathology analysis was performed after 24 hours and 7 days, respectively, from the intratracheal instillation of a solution containing 0.5, 2.5, or 5 mg/kg IOSi-NPs. The pathological micrographs of lungs derived from rats collected after the intratracheal instillation with a solution containing 0.5 mg/kg and 2.5 mg/kg IOSi-NPs show that the lung has preserved the architecture of the control specimen with no significant differences. However, even at concentrations of 5 mg/kg, the effect of IOSi-NPS on the lungs was markedly reduced at 7 days posttreatment.

## 1. Introduction

In recent years, an increasing interest has been registered for developing the field of nanotechnology. Due to the outstanding physicochemical properties that nanoparticles exhibit, the number of applications involving these nanomaterials is increasing continuously. Nowadays, nanoparticles can be found in toothpastes, sunscreens, or food products [1, 2]. The unique chemical, physical, optical [3, 4], electronic [3, 5], and magnetic [3, 6] properties show that nanoparticles could be used in biotechnology and biomedicine. The size of these nanoparticles facilitates their use in engineering of surfaces and in creating functional nanostructures [3]. Among the many types of nanoparticles, superparamagnetic iron oxide nanoparticles (SPIONs) have been already used for several* in vivo* applications, showing promising results. They were used as contrast enhancement in magnetic resonance imaging [7–9], for tissue repair [7, 10, 11], for drug delivery in tumour therapy [7, 12, 13], for stem cell tracking [7, 14], or as heat mediators in hyperthermia treatments [15]. In order to be used for these types of applications, the nanoparticles biocompatibility could be increased by adding a silica shell. Due to the biocompatible properties, silica is less likely to degrade in a biological environment [16, 17].

Cancer is a major health problem worldwide, being responsible for one in four deaths in the United States, according to the American Cancer Society [18]. In this context, researchers have tried to find new innovative ways of administering the treatment more efficiently. One of the major problems that arise during the targeted administration of drugs, most commonly used for cancer treatments, is the nonspecificity of the drug towards the pathological site [3]. Thus, a large dose of medicine is required in order for the treatment to be effective and the required local concentration to be achieved. The result consisted of the fact that besides the damaged cells, surrounding healthy cells could be also affected. Therefore, to overcome this impediment, researchers have focused their attention on developing techniques of magnetic targeting using superparamagnetic nanoparticles. They could be used as drug carriers, being guided by an external magnetic field, thus insuring an effective treatment [3, 19]. Wang et al. showed that the iron oxide nanoparticles coated with silica can be targeted to a specific objective via the antibody-antigen recognition by conjugation of antibodies to the outer silica surface [20]. Coating the iron oxide nanoparticles with various polymers such as silica, dextran, PEG, and HSA can prevent their uptake by organs. According to previous studies [21], the silica layer on the surface of iron oxide nanoparticles increases biocompatibility and stability against chemical degradation and can thus be used in applications such as bioseparation, genomic DNA isolation, or to control the drug release for several hours to days or months.

Another area that has attracted the interest of researchers and doctors alike is the field of stem cell treatment. Many efforts are made in order to personalize treatments by administering stem cells or genetically modified cells. Therefore, it is of great importance to trace the transplanted or injected cells and to assess their engrafting efficiency and functional ability. For this purpose, SPIONs are being considered as potential candidates [3, 22].

Although the biocompatibility of iron oxide nanoparticles has been demonstrated, previous studies showing that once the nanoparticles come in contact with a biological medium, their surface becomes covered with different types of proteins [7, 23], some SPIONs with core sizes between 3 and 6 nm, coated with dextran, being approved for MRI in patients [3, 24, 25], their biological activity can increase, causing potential toxic interactions [26]. It has been established that exposure to nanoparticles by people working in automobile, aerospace, or paint industries [27–32] can lead to major health problems, including neurotoxicity [7]. In this context, further studies are needed in order to establish the effects on the human body by inhaled nanoparticles.

The goal of this study was to prepare iron oxide-silica nanoparticles from a mixture of ferrous chloride tetrahydrate and ferric chloride hexahydrate dropped into a silica xerogel composite. The structure and morphology of the synthesized maghemite nanoparticles into the silica xerogel were analysed by X-ray diffraction (XRD) measurements, scanning electron microscopy (SEM) equipped with an energy dispersive X-ray (EDAX) spectrometer, and transmission electron microscopy (TEM). The biocompatibility of iron oxide nanoparticles embedded in a silica matrix was assessed by cell viability and cytoskeleton analysis. The biocompatibility of the iron oxide nanoparticles was evaluated using* in vitro* and* in vivo* assays, consisting in the quantification of HepG2 cells viability. On the other hand, histological evaluation of the effect caused by the obtained nanoparticles on the lungs of male Brown Norway rats after a single intratracheal instillation of a solution containing various concentrations of IOSi-NPs was performed in order to clarify the controversial toxicity of these nanoparticles.

## 2. Materials and Methods

### 2.1. Materials

Ferrous chloride tetrahydrate (FeCl_2_·4H_2_O), ferric chloride hexahydrate (FeCl_3_·6H_2_O), chlorhidric acid (HCl), ethanol, and tetraethylorthosilicate (TEOS) with 99.999% purity were purchased from Sigma-Aldrich and lead nitrate [Pb(NO_3_)_2_] with 99.5% purity was purchased from Merck.

### 2.2. Synthesis of Iron Oxide Nanoparticles in Silica Matrix

The mixture of ferrous chloride tetrahydrate (FeCl_2_·4H_2_O) in 2 M HCl and ferric chloride hexahydrate (FeCl_3_·6H_2_O) with the ratio Fe^2+^/Fe^3+^ = 1/2 was dropped into a silica xerogel composite under vigorous stirring for about 1 hour. The starting solution of silica xerogel composite was prepared by mixing tetraethylorthosilicate, water, and ethanol [33]. The water to TEOS and ethanol to TEOS mole ratios were 11.67 : 1 and 4 : 1. The gel was formed at room temperature under vigorous stirring. The formed gel based on iron oxide and silica was dried at room temperature. The final product was ground to form a fine powder. The obtained nanocomposite powder was then heat treated at 400°C in an oven, for 24 hours (iron oxide silica nanoparticles, IOSi-NPs).

### 2.3. Characterization

The X-ray diffraction measurements were recorded using a Bruker D8 Advance diffractometer, with nickel filtered CuK_*α*_ (*λ* = 1.5418 Å) radiation and a high efficiency one-dimensional detector (Lynx Eye type) operated in integration mode. The diffraction patterns were collected in the 2*θ* range 20°–70°, with a step of 0.02° and 34 s measuring time per step.

Transmission electron microscopy (TEM) studies were carried out using a FEI Tecnai 12 equipped with a low dose digital camera from Gatan. The specimen for TEM imaging was prepared by ultramicrotomy in order to obtain thin sections of about 60 nm. The powder was embedded in an epoxy resin (polaron 612) before microtomy. The particle size was measured by the SZ-100 Nanoparticle Analyzer (Horiba) using dynamic light scattering (DLS). The signal obtained from the scattered light is fed into a multichannel correlator that generates a function used to determine the translational diffusion coefficient of the particles analysed. The Stokes-Einstein equation is then used to calculate the particle size. Scanning electron microscopy (SEM) study was performed with a FEI Quanta Inspect F scanning electron microscope equipped with an energy dispersive X-ray attachment. The magnetic properties of the samples were measured using a superconducting quantum interference device (MPMS magnetometer) at room temperature.

### 2.4. Cell Cultures and Conditions

The hFOB 1.19 osteoblasts cells line and the HepG2 cells were purchased from American Type Culture Collection (ATCC CCL-121, Rockville, MD, USA). The cells were routinely maintained in Dulbecco's modified Eagle's medium (Sigma-Aldrich) supplemented with 10% fetal bovine serum (Sigma-Aldrich) and 1% antibiotic antimycotic solution (including 10,000 units penicillin, 10 mg streptomycin, and 25 *μ*g amphotericin B per mL, Sigma-Aldrich) at 37°C in a humidified atmosphere of 5% CO_2_. The cultured cells were loaded on iron oxide-silica nanocomposite discs at a seeding density of 5 × 10^4^ cells/cm^2^ in 24-well plates. Cells cultured in 24-well plates at the same seeding density were used as control.

### 2.5. *In Vitro* Cytotoxicity Assay

The viability of the cells was determined by the tetrazolium salt test [32]. The medium from each well was removed by aspiration, the cells were washed with 200 *μ*L phosphate buffer solution (PBS)/well, and then 50 *μ*L (1 mg/mL) of 3-(4,5-dimethylthiazol-2-yl)-2,5-diphenyltetrazolium bromide (MTT) solution was added on each well. After 2 hours of incubation, the MTT solution from each well was removed by aspiration. A volume of 50 *μ*L isopropanol was added and the plate was shaken to dissolve the formazan crystals. The optical density at 595 nm, for each well, was then determined using a Tecan multiplate reader (Tecan GENios, Grödic, Germany). The absorbance from the wells of cells cultured in the absence of ceramic discs was used as the 100% viability value.

### 2.6. Analysis of the Actin Cytoskeleton

The HepG2 cells were cultured in Dulbecco's Modified Eagle's Medium (DMEM) supplemented with 10% fetal bovine serum and 100 U/100 *μ*g/mL penicillin/streptomycin [34] (purchased from Invitrogen Co. (Carlsbad, CA)). After 24 hours of treatment, the medium was removed and the cells were fixed in 4% paraformaldehyde for 20 minutes at a temperature of 4°C and permeabilized with 0.5% Triton X-100 for 1 hour. The cells were washed with PBS five times. After being washed, the cells were incubated for 45 min in PBS containing FITC-phalloidin (20 *μ*g/mL, Sigma-Aldrich, St. Louis, MO). The nuclei of cells were counterstained with DAPI-4′-6-diamino-2-phenylindole (2 *μ*g/mL) for 3 min and rinsed with PBS. Mounted slides were visualized and photographed using a fluorescence microscope (Olympus BX51).

### 2.7. Animals

Male Brown Norway rats (weighing ∼300 ± 10 g) were purchased from the National Institute of Research and Development for Microbiology and Immunology “Cantacuzino,” Bucharest. The rats were housed in an environment controlled for temperature (22 ± 2°C), light (12 h light/dark cycles), and humidity (60 ± 10%). The animals were maintained under specific pathogen-free conditions in accordance with NIH Guide for the Care and Use of laboratory Animals.

### 2.8. Intratracheal Instillation

After acclimatization for one week, the rats were randomly divided into four groups (*n* = 4 per group) to receive vehicle control (saline, 0.9% NaCl, 0.3 mL) or instillation of a solution containing 0.5, 2.5, or 5 mg/kg IOSi-NPs. The rats were anesthetized with sodium methohexital (35 mg/kg, intraperitoneally) and placed on an inclined restraint board before instillation with saline suspension or IOSi-NPs. The IOSi-NPs were suspended in physiological saline solution. Before intratracheal instillation, the IOSi-NPs suspension was ultrasonicated for 30 min. The instillation was performed using a nonsurgical intratracheal instillation method [35] adapted from Hatch et al. [36]. The rats were anesthetized by ether. According to Bai et al. [35], a ball tripped needle was maneuvered through the epiglottis, after which contact with the tracheal rings provides confirmation that the needle is, in fact, within the trachea. Afterwards, an injector with 100 *μ*L physiological saline or IOSi-NPs suspension was inserted into the ball tripped needle. To allow the fluid to drain into the respiratory tree, after the physiological saline or IOSi-NPs suspension gently instilled into the trachea, the animal was maintained in an upright position for 5 min. The rats were euthanized and sacrificed after 24 h and one week from the instillation, according to the Guide for the Care and Use of Laboratory Animals. All animals were humanely treated and were monitored for any potential suffering.

### 2.9. Histological Examination

Histopathology analysis was performed in Floreasca Emergency Hospital, Bucharest, Romania. The lungs derived from the rats in the control group and the treated groups were fixed with 10% neutral buffered formalin and processed using routine histological techniques. After paraffin embedding, 3 *μ*m sections were cut and stained with hematoxylin and eosin (H and E) for histopathologic evaluation. The morphological changes were observed under the microscope [37].

## 3. Results and Discussions

The IOSi-NPs have been widely used for environmental, biological, and medical applications but their potential toxicity at nanometric scale provides a growing concern about the risk factor for human health. In order to assess the risks of IOSi-NPs, the objective of this study was to evaluate and compare the pulmonary responses induced in rats after intratracheal instillation of suspensions containing various concentrations of IOSi-NPs.

Before carrying out the study on evaluating and comparing pulmonary responses induced in rats after intratracheal instillation of suspensions containing various concentrations of IOSi-NPs, characterization of the synthesized ultrafine IOSi-NPs particles was performed using XRD analysis, EDAX analysis, scanning electron microscopy, and transmission electron microscopy.

**Figure 1:**
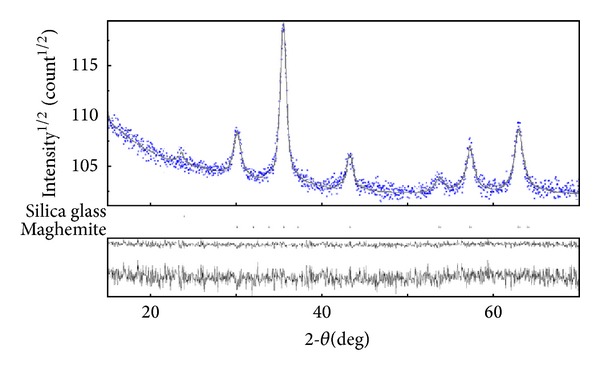
Experimental (blue), calculated (solid line gray), and difference plot (lover line) of *γ*-Fe_2_O_3_ and silica.

Good pattern fit has been achieved by the Rietveld method using MAUD (material analysis using diffraction) [38]. The XRD analysis of iron oxide-silica nanocomposite Rietveld refinement of X-ray diffraction patterns revealed a phase mixture of maghemite and amorphous silica. The comparison between the experimental and calculated data obtained with the joint Rietveld refinement of the sample is presented in Figure 1.

The experimental data in blue and the calculated data are represented by a grey line. Vertical lines represent the positions of diffraction lines of maghemite and amorphous silica. The line below the grey plot is the difference profile. It resulted in that each sample is constituted of spherical nanocrystallites. The diffraction peak at about 2*θ* = 23 is related to amorphous silica. Another diffraction peak corresponding to the Miller indices value (hkl) of (220), (311), (400), (422), (511), and (440) agrees with the cubic structure of maghemite in Fd3m space group (ICSD-PDF no. 79196) with a lattice parameter of 8.35 Å. The XRD showed a slight broadening of the diffraction lines which can be interpreted in terms of small sized crystallites [39]. The calculated particle size of maghemite silica nanocomposites was estimated at around 12.5 nm. Based on the XRD data refinement, the formation of single-phase spinel cubic structure belonging to the Fd3m space group has been confirmed. The progress of the Rietveld refinement for the sample is monitored by a number of agreement parameterssuch as the weighted profile *R*_wp_ index and the “goodness of fit” (*χ*) index which is the ratio of *R*_wp_ over the statistically expected *R*_exp⁡_.

On the other hand, in the Rietveld method, *R*_Bragg_ is useful because it depends on the fit of the structural parameters and not on the profile parameters. The final *R* factors [40] obtained from the analysis of the iron oxide-silica nanocomposites were given by *R*_wp_ = 1.0058 (%) and *R*_exp⁡_ = 0.9530 (%). The resulting Bragg *R*-factor and chi squared (*χ* ^2^) were 0.58 and 1.1138, respectively. Theoretical values of the *R* factors obtained for maghemite-silica nanocomposites are in good agreement with the theory of Toby [41].

**Figure 2:**
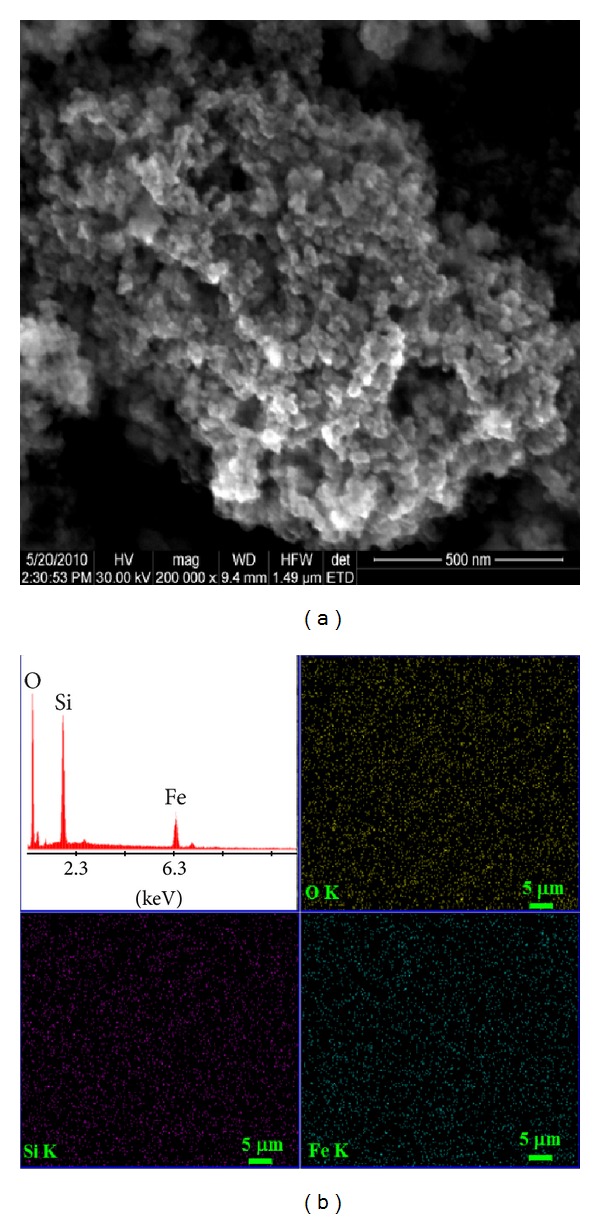
SEM micrographs (a) and elemental maps of maghemite-silica nanocomposite.

Information about the size and typical shape of the IOSi-NPs obtained after heat treatment at 400°C of the obtained initial nanocomposites powder based iron oxide and silica was provided from SEM analysis. SEM image of maghemite-silica nanocomposites showed very small particle sizes and uniform spherical shapes (Figure 2(a)). EDAX spectrum and elemental maps (Figure 2(b)) of Fe, O, and Si for the maghemite-silica nanocomposites are also presented. The uniform distributions of Fe, O, and Si could be observed.

**Figure 3:**
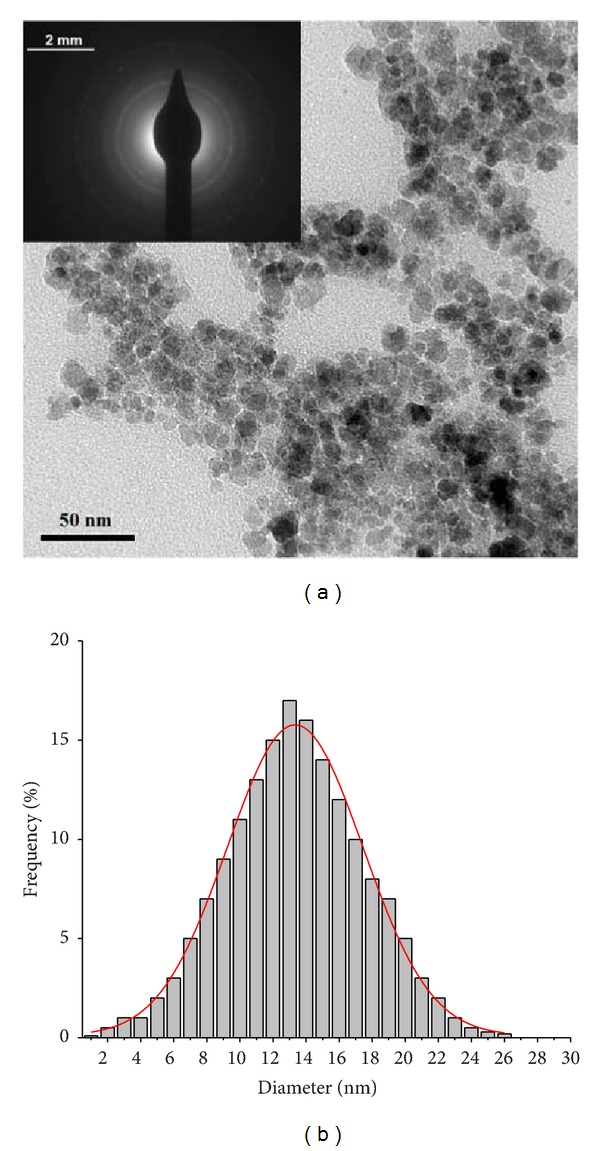
TEM micrograph (high magnification at 180000x) showing the spherical IOSi-NPs, selected area electron diffraction (SAED), and size distribution.

The morphological investigation of iron oxide-silica nanoparticles was performed using transmission electron microscopy (TEM).  Figure 3 shows the TEM image of IOSi-NPs, the selected area electron diffraction (SAED), and size distribution of the particles. Details observed at high magnification (180000x) show monodisperse IOSi-NPs with spherical shape and a monomodal particle size distribution. The average size deduced from the particle size distribution is 13.7 ± 0.6 nm, which is in good agreement with XRD analysis.

**Figure 4:**
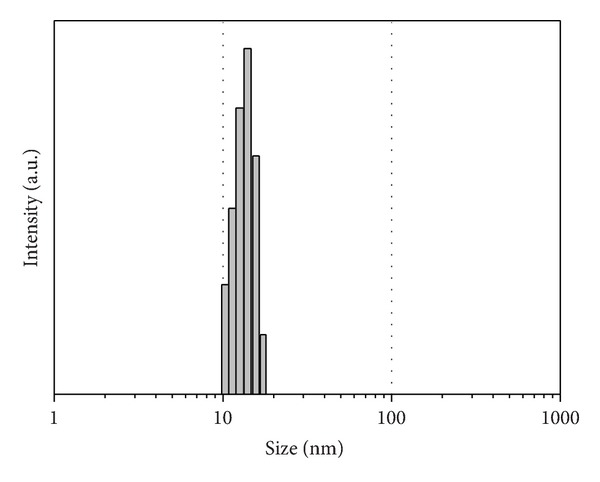
DLS showing mean average size of maghemite-silica nanoparticles.

Dynamic light scattering known as Quasi-Elastic Light Scattering was used to determine the size distribution profile of small maghemite-silica particles in suspension. The maghemite-silica nanoparticles observed by DLS are monodisperse in water (Figure 4). The size of IOSi-NPs deduced from TEM images is consistent with the DLS size profiles (14 nm).

**Figure 5:**
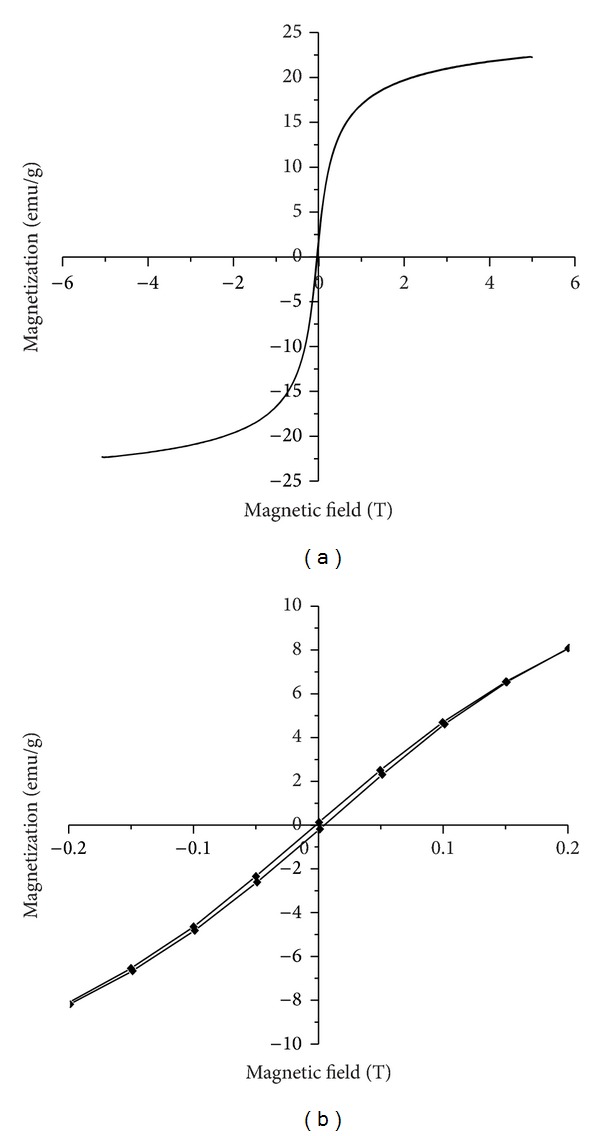
M-H curves of IOSi-NPs at room temperature in full scale (a) and in extended scale (b).

Magnetic measurements of the iron oxide-silica nanoparticles were carried out using vibrating sample magnetometer (VSM). Magnetization curves (M-H loop) for the IOSi-NPs obtained after heat treatment at 400°C measured at room temperature are presented in Figure 5.

According to Dormann et al. [42], iron oxide-silica nanoparticles keep the superparamagnetic characteristic of the iron oxide nanoparticles. From hysteresis measurements, the zero coercivity fields of IOSi-NPs can be considered equal to 0 Oe at 300 K. The very little value of coercivity fields observed in Figure 5(b) can be attributed to the fact that iron oxide nanoparticles in silica matrix do not have the ability to rotate freely. In agreement with Im et al. [43], the magnetic domain sizes of iron oxide nanoparticles in silica matrix can be easily over the limit of superparamagnetism. According to previous studies [43, 44], the silica colloids at room temperature exhibit a very small coercivity. On the other hand, the value of saturation magnetizations, Ms, was about 22.29 emu/g. In previous studies, Pareta et al. [45] have attributed the low saturation magnetization of iron oxide in silica matrix to the diamagnetic contribution of the SiO_2_ shells covering the iron oxide nanoparticles, thus weakening the magnetic moment for iron oxide/SiO_2_ nanoparticles due to the occurrence of thicker shells.

**Figure 6:**
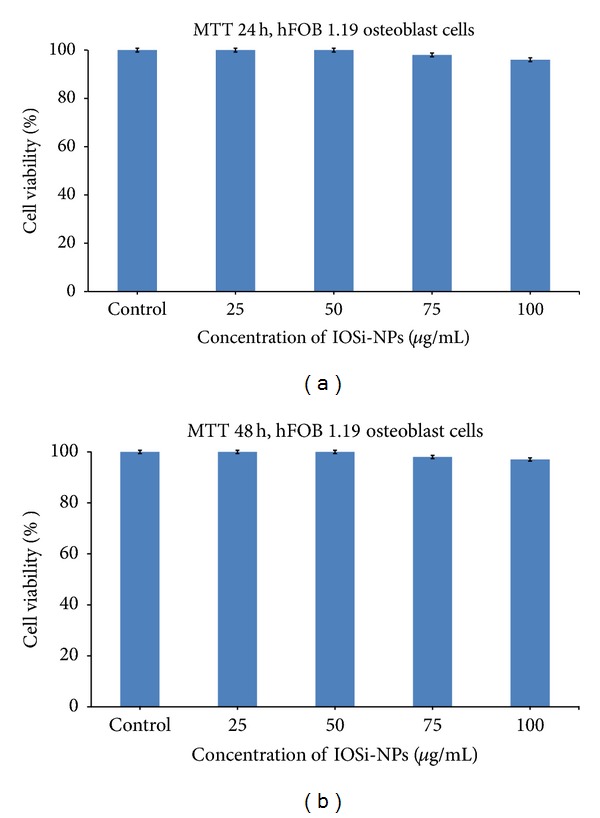
Cell viability data assessed by a MTT assay for hFOB 1.19 osteoblast cells incubated for 24 h (a) and 48 h (b) with the IOSi-NPs at various concentrations (25, 50, 75, and 100 *μ*g/mL).

In order to quantitatively evaluate the hFOB 1.19 osteoblast cell viability, we performed MTT assay (Figure 6). The cell viability of hFOB 1.19 osteoblasts cells in the presence of various concentrations of IOSi-NPs (concentrations from 25 to 100 *μ*g/mL) after 24 h indicated that osteoblast viability was similar in the presence of IOSi-NPs compared to the control, as shown in Figure 6(a). After exposure to increasing concentrations from 25 to 100 *μ*g/mL of IOSi-NPs for 48 h, the cell viability of hFOB 1.19 osteoblasts cells is not influenced by the presence of IOSi-NPs (Figure 6(b)).

In the present study, after 24 h and 48 h from exposure with IOSi-NPs, there could not be observed higher values of cell viability in the presence of IOSi-NPs (concentrations from 25 to 100 *μ*g/mL) compared to the control. It was thus proved that the IOSi-NPs remain dispersed in cell culture, keeping bioactivity for all concentrations from 25 to 100 *μ*g/mL [45]. The cytotoxicity test by MTT assay, using hFOB 1.19 osteoblasts cells, has indicated that the studied IOSi-NPs were nontoxic to the cells.

**Figure 7:**
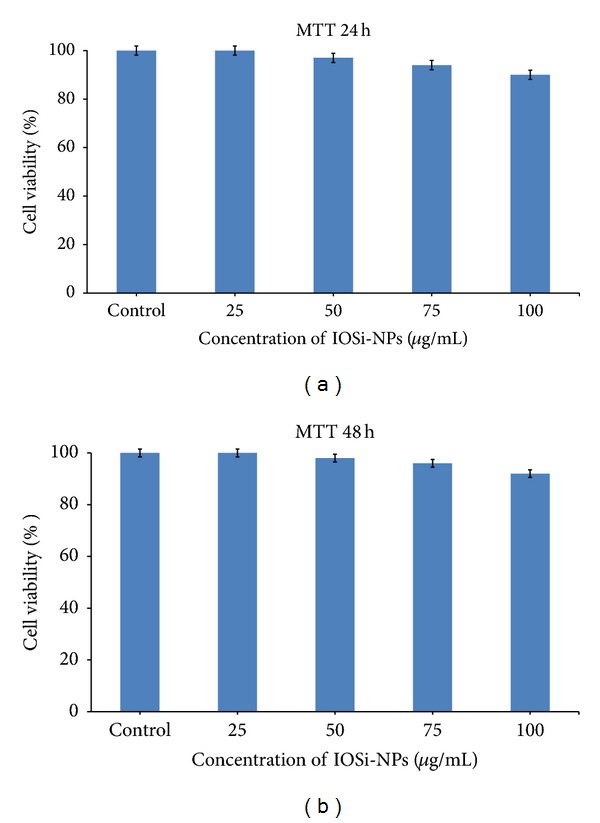
Cell viability data assessed by a MTT assay for HepG2 cells incubated for 24 h (a) and 48 h (b) with the IOSi-NPs at various concentrations (25, 50, 75, and 100 *μ*g/mL).

On the other hand, we tested the cell viability of HepG2 cells in the presence of various concentrations of IOSi-NPs and the expression of F-actin in HepG2 cells adhered on IOSi-NPs nanoparticles. After exposure to increasing concentrations from 25 to 100 *μ*g/mL of IOSi-NPs for 24 h and 48 h, the cell viability of HepG2 cells was measured using MTT assay (Figure 7). In order to investigate the cell viability, it is relevant to note that in all the studies, the cells are not influenced by the presence of IOSi-NPs, preserving a good morphology adhesion. When the concentration of IOSi-Nps increased, the cell variability decreased from 100% (25 *μ*g/mL) to 90% (100 *μ*g/mL) after 24 h. After 48 h, the cell variability decreased to 92% when the IOSi-NPs concentration is equal to 100 *μ*g/mL [46]. Furthermore, after 48 hours of exposure with IOSi-NPs, we observed a tendency of linear increase of viability and proliferation (Figure 7).

This effect might be due to cells adaptation at interaction with IOSi-NPs nanocomposites. According to Weichsel et al. [47], the modeling and spatial organization of the actin cytoskeleton is a very active and increasingly sophisticated research.

The functions of the actin cytoskeleton are to mediate a variety of essential biological functions in all eukaryotic cells, including intra- and extracellular movement and structural support. In order to perform these functions, the organization of the actin cytoskeleton must be tightly regulated both temporally and spatially [48]. According to previous studies on the size effect on cell uptake in well-suspended, uniform mesoporous silica nanoparticles [49], facile synthesis of monodispersed mesoporous silica nanoparticles with ultra large pores and their application in gene delivery [50] and silica-based complex nanorattles as multifunctional carrier for anticancer drug [51] showed that for intracellular drug delivery and efficient therapy, an efficient cellular internalization of nanoparticles is necessary.

**Figure 8:**
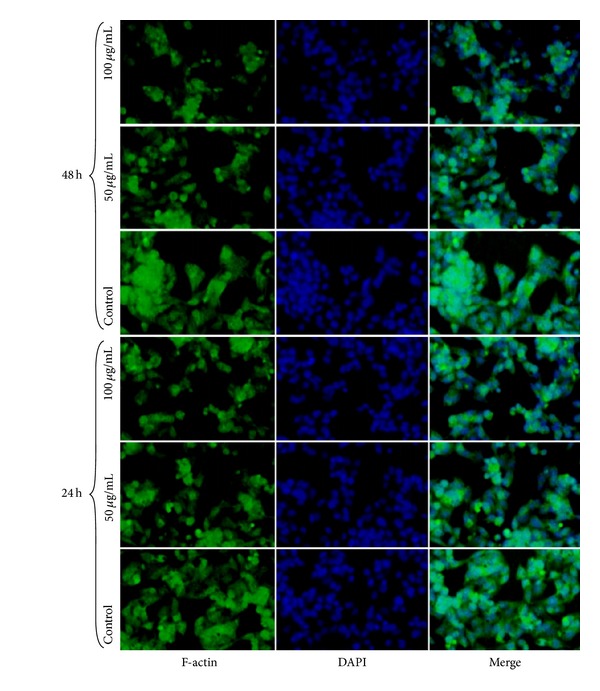
F-actin—images of F-actin stained by FITC-Phalloidin (green), DAPI—images of nuclei stained with DAPI (blue), and merge—the merged picture. The cells were cultured for 24 h and 48 h in the presence of 50 and 100 *μ*g/mL of IOSi-NPs.

For detection of F-actin, the major constituent of microfilaments (green), the cells were fixed and stained with FITC phalloidin (Figure 8). The cells were stained with 4,6-diamidino-2-phenylindole dihydrochloride (DAPI) in order to visualize cell nuclei.

After cells treatment with the IOSi-NPs, there could not be found any morphological changes with observable fragmented nuclei. Moreover, staining of F-actin and nuclear staining have shown that the assimilation of the nanoparticles did not have any effect on nuclear morphology nor on the cytoskeleton of transfected cells.

**Figure 9:**
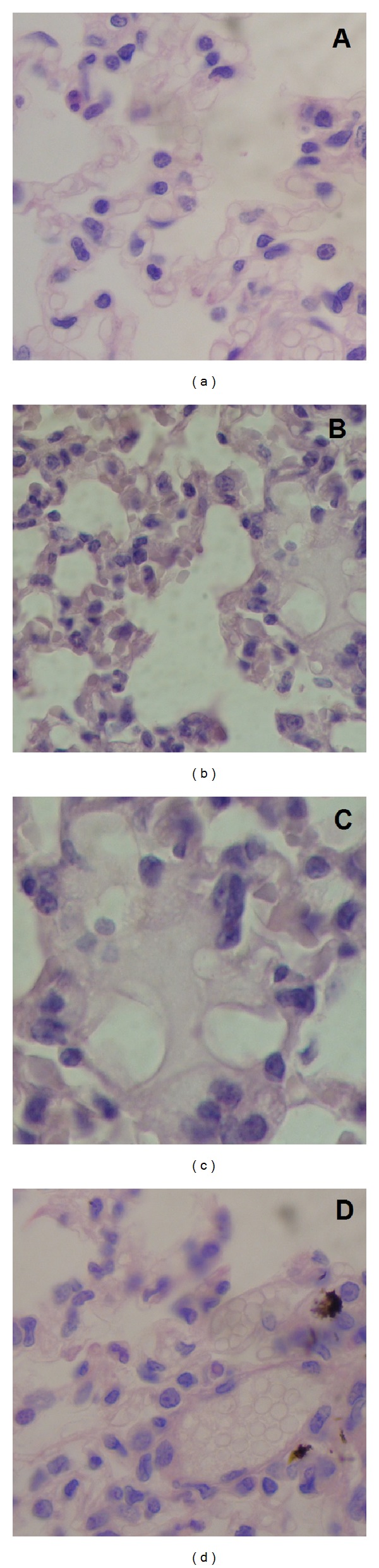
Light optical image of the lung at 24 h after intratracheal instillation of IOSi-NPs in rats at various concentrations. The reference sample is also presented (a). Lung after 24 hours: control (a), 0.5 mg/kg (b), 2.5 mg/kg (c), and 5 mg/kg (d).

**Figure 10:**
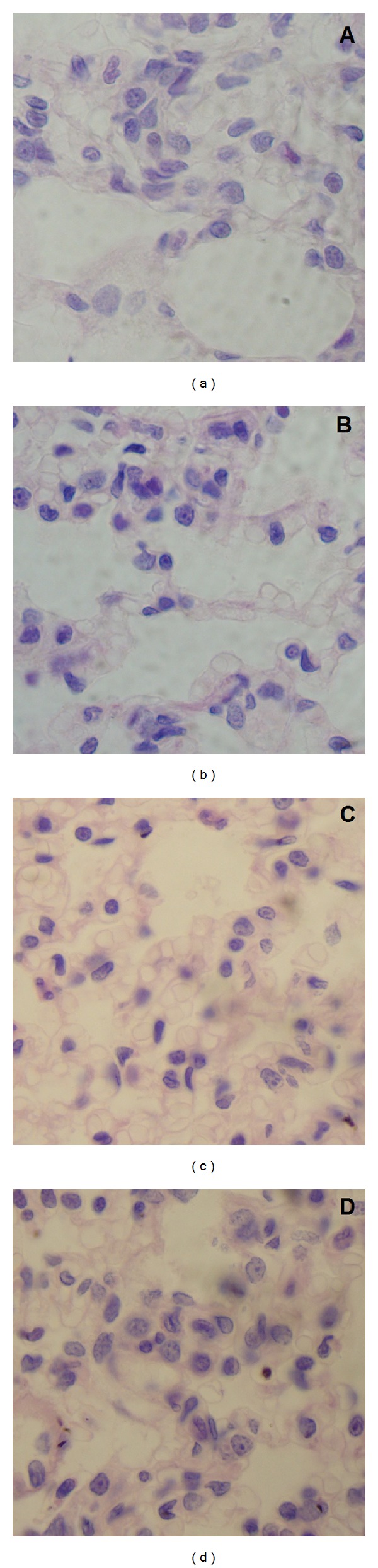
Light optical image of the lung at 7 days after intratracheal instillation of IOSi-NPs in rats at various concentrations. The reference sample is also presented (a). Lung after 7 days: control (a), 0.5 mg/kg (b), 2.5 mg/kg (c), and 5 mg/kg (d).

**Figure 11:**
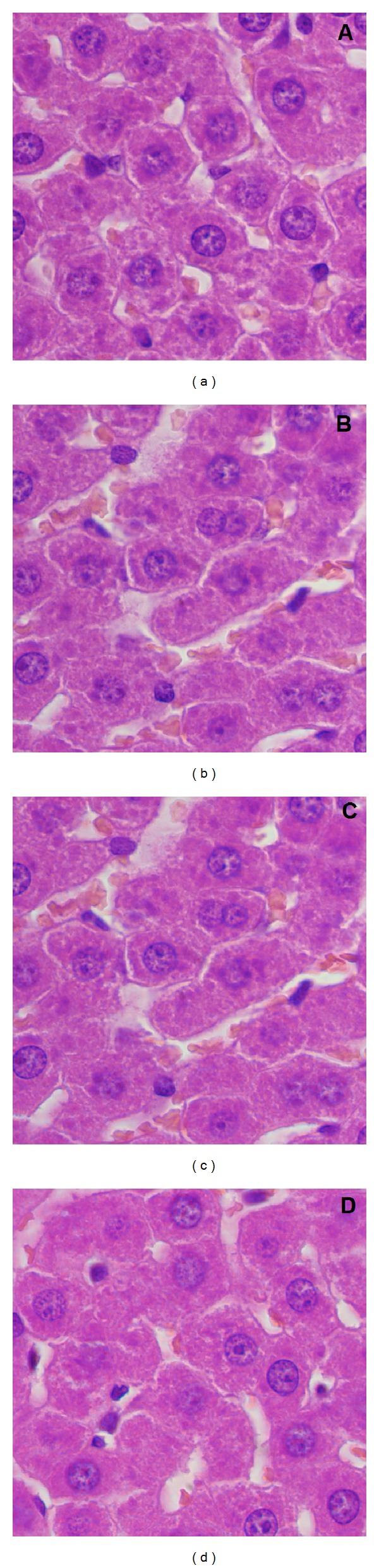
Light optical image of the liver at 24 h after intratracheal instillation of IOSi-NPs in rats at various concentrations. The reference sample is also presented (a). Liver after 24 hours: control (a), 0.5 mg/kg (b), 2.5 mg/kg (c), and 5 mg/kg (d).

**Figure 12:**
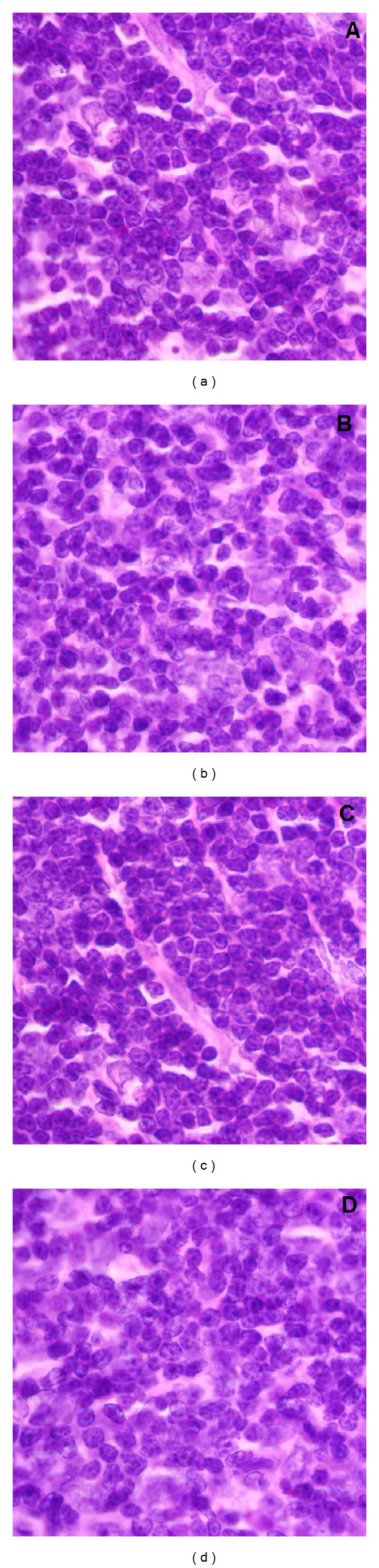
Light optical image of the spleen at 24 h after intratracheal instillation of IOSi-NPs in rats at various concentrations. The reference sample is also presented (a). Spleen after 24 hours: control (a), 0.5 mg/kg (b), 2.5 mg/kg (c), and 5 mg/kg (d).

According to previous studies [52, 53], at this stage, little is known about the possible interactions of nanomaterials with biological systems. Development of new nanomaterials may lead to major health problems induced by frequent exposure to these airborne nanoparticles. From this point of view, the potential toxic effects of IOSi-NPs by intratracheal instillation are discussed. Although it has been shown that the iron oxide and silica nanoparticles have an excellent potential for biomedical applications, there is not an understanding of their potential systemic toxicity. According to the previous studies of Drobne [54] and Seaton [55], the particular nanoscale properties are likely to affect not only the chemistry and physics but also their behaviour in biological systems. The toxicity evaluation after 24 h and 7 days from intratracheal instillation of various concentrations of IOSi-NPs in rats was performed by histopathological analysis (Figures 9–12). All animals survived the administration of IOSi-NPs on all the tested concentrations and did not show any sign of discomfort (lethargy, nausea, vomiting, or diarrhea) during the whole duration of the experiment. The histopathological assessment of the selected tissues such as lung and liver was conducted.

The toxicity evaluation of the lung after 24 h from intratracheal instillation of various concentrations of IOSi-NPs in rats was observed by histopathological investigations (Figure 9). After 24 h from intratracheal instillation, the rats showed particle-induced modifications that were dependent on the concentrations used. After 24 h from the intratracheal instillation with 0.5 mg/kg of IOSi-NPs, the lung parenchyma of the rats showed preserved alveolar architecture with rare macrophages in the alveolar septa. We could see that the pathological micrographs of lung in rats after the intratracheal instillation with 0.5 mg/kg dose of IOSi-NPs (Figure 9(b)) show that the lung has preserved the architecture of the control specimen (Figure 9(a)), with no significant differences. After the intratracheal instillation of the rats with a 2.5 mg/kg (Figure 9(c)) dose of IOSi-NPs, the lung parenchyma of the rats showed preserved alveolar architecture with rare macrophages in the alveolar septa, discreet anisokaryosis, and anisochromia of type II pneumocytes with rare nucleoli. Lung parenchyma of the specimen after intratracheal instillation of IOSi-NPs in rats at concentration of 5 mg/kg (Figure 9(d)) showed preserved alveolar architecture with macrophages in the alveolar septa, discreet anisokaryosis, and anisochromia of type II pneumocytes, with chromocenters and nucleoli. In the lung parenchyma, focal ectatic capillaries were also observed in the alveolar septa.

However, the effect of IOSi-NPS on lungs, even for concentration of 5 mg/kg, was markedly reduced after 7 days of treatment. After 7 days from intratracheal instillation of the rats with 5 mg/kg IOSi-NPs (Figure 10(d)), we observed that the lung parenchyma show preserved alveolar architecture with rare macrophages, discreet anisokaryosis, and anisochromia of type II pneumocytes, with rare chromocenters and nucleoli (Figure 10(d)). On the other hand, the pathological micrographs of lungs in rats after the intratracheal instillation with 0.5 mg/kg and 2.5 mg/kg dose of IOSi-NPs (Figures 10(b)-10(c)) show that the lung has preserved the architecture of the control specimen (Figure 10(a)) with no significant differences.

According to the literature [56], the liver is the first organ exposed to nanoparticles that are capable to enter into the circulation after intratracheal instillation because it is the major organ for biotransformation of toxins. The liver examination after intratracheal instillation of the rats with various concentrations of IOSi-NPs (Figure 11) indicates that the liver has preserved the architecture of the control specimen (Figure 11(a)) with no significant differences even for values of 5 mg/kg.

After microscopic examination of the liver and spleen 24 h from intratracheal instillation of IOSi-NPs in rats, we can see that none of the organs other than the lung revealed any related toxic response, in good agreement with previous studies [57].

Previous studies [58, 59] suggested that smaller sized nanoparticles could initiate lung injury such as the inflammation in lung, which induced endothelial-epithelial damage and subsequent infiltrated leukocytes that allowed large amounts of smaller nanoparticles to pass easily into circulation. Moreover, in the lung injury, the size and concentrations of nanoparticles play a major role.

Iron oxide nanoparticles were developed rapidly and because of their superparamagnetic characteristics, they are of considerable interest for potential applications in biology and medicine, magnetic target drug delivering, and environmental catalysis. According to previous studies of Wu et al. [60], the surface functionalized magnetic iron oxide nanoparticles are a kind of novel functional material, which has been widely used in the biotechnology and catalysis. The present studies enrol to investigate the interaction of these nanoparticles with biological systems. The aim of this study was to evaluate the potentially toxic effects of IOSi-NPs on the lungs, spleen, and liver of rats after being exposed for 24 h and 7 days, respectively, to a single intratracheal instillation of solutions containing concentrations of 0.5, 2.5, or 5 mg/kg.

Consistent with other reports examining various materials like CeO_2_ [61, 62], titanium dioxide [63], silica [64], and copper [65] nanoparticles, our data suggest that IOSi-NPs are not capable of translocating from the lungs to the liver via the circulatory system at all the tested concentrations. The histopathological appearance of the liver after intratracheal instillation of IOSi-NPs in rats shows that the different pathological alterations such as enlargement of hepatocytes, hydropic degeneration of hepatocytes, nuclear enlargement, and dilatation of the sinusoids were not observed at concentrations of 0.5, 2.5, and 5 mg/kg.

## 4. Conclusions

The purpose of our research focuses on the development of various strategies of synthesis and control of the structure and magnetic properties of surface functionalized iron oxide nanoparticles and their corresponding applications. Iron oxide nanoparticles were encapsulated in a silica matrix. Silica coated magnetic nanoparticles have been previously synthesized by adding into a silica xerogel composite the mixture of ferrous chloride tetrahydrate and ferric chloride hexahydrate. A systematic study of the formation of iron oxide coated with silica was made. The IOSi-NPs characterized by various techniques were found to have a narrow size distribution with an average size deduced from TEM measurements of around 13.7 ± 0.6 nm in good agreement with the size deduced from the XRD analysis that was estimated at around 12.5 nm.

In terms of the viability, it can be noted that in all the studies, the cell viability was not influenced by the presence of IOSi-NPs, preserving a good morphology adhesion.

After microscopic examination of the lungs, liver, and spleen after 24 h from intratracheal instillation with doses of 0.5 mg/kg, 2.5 mg/kg, and 5 mg/kg IOSi-NPs in rats, we could see that none of the organs other than the lung revealed any toxic response. On the other hand, the effect of IOSi-NPS on lung, even for a concentration of 5 mg/kg, was markedly reduced after 7 days of treatment.

In conclusion, we demonstrated that intratracheal exposure with doses containing 0.5 mg/kg, 2.5 mg/kg, and 5 mg/kg IOSi-NPs did not initiate acute lung injury and that the synthesized nanoparticles with sizes around 12.5 nm are not able to enter into the circulatory system.

In this study we tried to answer some questions of the currently pressing problems regarding the toxicity of nanoparticles and their behaviour* in vitro* and* in vivo*. For a better understanding of the behaviour of these nanoparticles on health more complex studies will have to be carried out in the future on toxicity induced after intratracheal instillation of fine and ultrafine particles.
